# Remarkable Case of Oneirodynia

**Published:** 1817-02

**Authors:** Aloysius Polydore


### Journal and Review.

VOL. III.] FEBRUARY, 1817. [NO. 14.

### PART I.

### ORIGINAL COMMUNICATIONS.

—— quid utile ——

### *REMARKABLE CASE of ONEIRODYNIA.*

By Aloysius Polydore, M. D.

" A great perturbation in Nature ! to receive at once the benefit of sleep, and do the effects of watching !"—Shakesp.

A BOY, 10 years of age, whose mother had long been afflicted with an obstinate head-ache, and whose aunt was subject to epileptic attacks, was seized in the beginning of February, 1793, with a clonic convulsion or tremor of his knees, which was succeeded by a sudden fall to the ground; by head-ache; and, finally, by sleep. This train of symptoms had frequently recurred in the above order, when he began to be troubled with a kind of alternate dilatation and contraction in his left hand, a crepitus being perceptible in the same wrist. In the ensuing April, to the foregoing symptoms was added a pain in the right hip, which soon shifted to the knee, and afterwards to the great toe. In the course of a few days this pain was translated to the left side, attacking the hip, knee and toe in succession as before; but this paroxysm was always preceded by a pain in the middle, the ring, or the fore-finger of the *right* hand. At first, these paroxysms of pain lasted for forty-eight hours, without intermission:—Shortly afterwards, they only attacked the patient every second day, after sunset. Lastly, the paroxysm came on every fourth day, with tremors of the lower extremities.

After being bled, purged, and put into the warm bath, he used some anthelmintics, and obtained a respite of ten days from the abovementioned symptoms. At the expiration of this period he was attacked with slight, but freqnent wandering pains, which, towards the beginning of May, were followed by head aches, and convulsive motions of the lips, eyes, and muscles of the chest. A short interval of good health was now the prelude to another train of symptoms. The foregoing attacks returned, and each paroxysm was followed by a short sleep, which was succeeded, in its turn, by a series of sudden gesticulations, and great garrulity, the subject of which was, the fear of an attack by the French. These symptoms used frequently, and without order or regularity, to commence and remit; when, all at once, the boy felt a violent stricture in his throat: his swallowing became impeded; he began to be convulsed with spasms; he danced, sang, gesticulated like a pugilist, and roared out with terror when ever so slightly touched. This was the account which I received when I first saw him.

On the 25th of June 1793, being sent for in the night to see the boy, I found him lying in bed. He readily recognized me, as indeed he did the others who surrounded him; and on my asking him how he felt? he answered rationally, that his hips and legs were painful, especially when pressed. His pulse was irregular, small, and hard; yet his breathing appeared natural and easy. As I was watching the pulse, I twice or thrice observed, that when the strokes became slow and obscure, his lower extremities were agitated with quick, convulsive vibrations. After a short apparent sleep, he began to gesticulate, and, in a pleasing accent, pronounced some unmeaning words, with a look highly expressive of internal satisfaction. He next exclaimed, that his leg was at liberty; and by degrees expressed his joy at the freedom of the other. He at length rose up; walked about in every direction on the bed, but never put his foot beyond the edge of it. He now danced, and recited with facility what he had seen, said or done, lately and long before. While thus employed, he put his hand on a cap, and, as he had been accustomed to do while in health, he threw it up towards the ceiling, seemingly with the intention of its remaining on a beam which traversed an empty space above the bed. A

servant who occasionally approached the bed, he attacked violently with his fists. In order that I might discover whether or not he could distinguish objects by the sight, I ordered the servant to touch him, and instantly start to one side, so as to elude his blows. Irritated by this, he dealt his blows in every direction in the air, and seemed anxiously to seek the object of his resentment. He now sprang towards a painting that was hanging on the wall; took it down; kissed it, and laid it by him in the bed, as though he wished it to sleep there. When it was taken away privately by the servant, he got up and repeatedly passed his hand over the place where it used to hang, for the purpose of finding it. He next sprang out of bed and began to beat all who came in his way with a pillow and also with his fists. He walked round the chamber without once running against the walls; and being desirous of making water, he readily found the pot de chambre, and after using it laid himself down to sleep on a chest with a pillow under his head; but finding, I suppose, the boards too hard, he spread under him some clothes that were hanging on a beam, and went tranquilly to sleep. On trying to feel his pulse, he resisted and drew back his hand: but perceiving that he was not secure in this way, he arose, put on a cloak with long sleeves, and folding them over one another, "Now, says he, laughing, touch me if you can." He began next to feel us all individually, and readily recognized his mother and a maid servant who were in the room. He took water out of a bottle into his mouth and spurted it over us. To try the state of the organs of taste, I mixed some scrapings of a cake of chocolate in water, which he took in his mouth from a cup; but instantly spat it out, saying it was decoction of bark. To try the olfactory nerves, I fired a piece of paper and placed it under his nose; he immediately cried out "that the house was in flames." That his nerves of sight were not in action, was proved by several circumstances, but particularly by the application of a torch, which he did not put away nor avoid when it was held and moved close to his open eyes. The loudest noises made close to his ears were disregarded as though he had been a statue, proving beyond doubt that the auditory nerves were insensible. In two hours he fell fast asleep, in which state he continued till the morning. On awaking, he remembered not an iota of what had passed. The next night, nearly a similar train of phænomena was developed; the same experiments made, and the same results obtained.

Three circumstances are here worthy of commemoration. He had by chance found, while in health, that the pulses of the radial artery were rendered indistinct by keeping the tendons of the wrist in motion. Accordingly when I attempted to feel them, he instantly began to move his fingers. Nor would he let the pulses of the temporal artery be counted. For having applied his hand to his temple while he made his teeth chatter, and thereby ascertained that the temporal pulse was rendered doubtful, he quickly moved his jaw upwards and downwards whenever I laid my fingers on the artery. At length, being tired by my persevering efforts, he said—"last night I prevented you with the cloak; but I shall shortly do the same with my fists." This circumstance is truly wonderful; for the boy had no recollection of the cloak business during the waking interval between the two nights. Taking his pillow now for a crucifix, he walked through the chamber, making his genuflexions, and repeating the intercalar verse "*Adoramus te Christe, &c.*" in imitation of those who prostrate themselves while meditating on the passion of Christ. It may here be remarked, that while the boy laboured under this singular affection, his sister, and another girl in the family, both about fourteen years of age, were somewhat similarly affected. In them the oneirodynia was always preceded by hysterical and spasmodic symptoms. They all three recovered, more by the efforts of Nature than the effects of remedies.

### *Observations, by J. G. Polydore, M. D.*

This singular disease appears evidently to have originated in some cerebral affection, since it was preceded both in the boy and in the two females, by spasms.* What the exact nature of this cerebral affection was, I am unable to say; but if I am allowed to hazard a conjecture I would suppose it originated, not from any *organic* lesion of the encephalon, but rather from a morbid or præternatural irritability of that organ or its nerves. The first remarkable phænomenon is, that the memory was not only unimpaired, but *unusually* clear. He remembered not alone what he had done in his waking hours, but even what he

---

* We do not entirely coincide with Dr. Polydore on this point. We think it much more probable, that the origin of the affection was in some part of the long and intricate line of the digestive organs, from whence it was propagated to the sensorium by one of those inexplicable sympathies which we recognize only by their effects.—Edit.

had done in the preceding paroxysm. The latter he could not remember when awake. It seems even that his understanding was improved, from the ingenious means which he used to prevent the radial and temporal arteries being felt.

It was the opinion of my uncle, the narrator of the case, that the boy was totally devoid of sight during the paroxysm; but there are some circumstances apparently inexplicable on this supposition. How, unless he could see, was he able to throw up his cap at the beam, and catch it on its fall, or find it on the ground? There are other indeed, and very cogent reasons for denying him the power of vision; such as the neither putting away, nor avoiding the torch that was held before his eyes; also his searching for the picture where it was not; and his being unable to find the servant. May we not explain the want of vision *occasionally*, by supposing that at those periods there was nothing in his ideas that guided him to the object, and that, in searching for the picture, the impression on his imagination and memory was more vivid than that transmitted by the sight; as it frequently happens that by a mechanical habit we search for a book on the desk or shelf where it used to be, though we know that it is not there. I would infer, therefore, that he only saw when his imagination prompted any idea to him, in the pursuit of which sight was necessary; and, on this account, I would suppose, that he did not see the torch, because his mind was intent on something else. The other senses, except that of hearing, were open to every impression, as is evident from the experiment made; but open only, I think, when something was supplied by the imagination. Altogether, the case is very remarkable, and capable of affording materials for reflection both to the physician and metaphysician.

*Edinburgh, August* 1, 1816.

---

